# Commentary: Iridoids derived from Valeriana jatamansi jones alleviates neuroinflammation and blood spinal cord barrier permeability after spinal cord injury by activating the Nrf2/HO-1 signaling pathway

**DOI:** 10.3389/fphar.2025.1699044

**Published:** 2025-11-06

**Authors:** Kai Zhang, Lei Wu

**Affiliations:** 1 Department of Neurobiology and Acupuncture Research, Key Laboratory of Acupuncture and Neurology of Zhejiang Province, The Third School of Clinical Medicine (School of Rehabilitation Medicine), Zhejiang Chinese Medical University, Hangzhou, China; 2 Department of Acupuncture, The Third Affiliated Hospital of Zhejiang Chinese Medical University, Hangzhou, China

**Keywords:** spinal cord injury, IRFV, blood spinal cord barrier (BSCB), Nrf2/HO-1, neuroinflammation

## Introduction

We carefully read the article by He Y et al. in Frontiers in Pharmacology. This article demonstrates that IRFV alleviates neuroinflammation and blood-spinal cord barrier (BSCB) injury after spinal cord injury (SCI) by activating the Nrf2/HO-1 pathway, providing a clear molecular mechanism for traditional Chinese medicine active ingredients (He et al., 2025). By targeting BSCB integrity beyond neuronal repair, this work reveals IRFV’s indirect role in neuroinflammation—a mechanism confirmed through complementary *in vivo* and *in vitro* models. Neuroprotection by IRFV was comprehensively confirmed through multiple approaches, including behavioral analysis (BBB score), histopathology (H&E), ultrastructural examination (TEM), apoptosis assessment (TUNEL), inflammatory cytokine quantification (ELISA), and barrier permeability evaluation (Evans Blue). We believe this study significantly contributes to SCI treatment research using traditional medicines. However, we offer suggestions to further strengthen the work.

### Increase the longitudinal analysis of time-dependent neuroprotection of IRFV after SCI

Studies have established that the inflammatory microenvironment and BSCB following SCI undergo dynamic temporal changes (Lee et al., 2019). Evidence indicates day 3 post-injury represents the acute inflammatory phase, while day 14 corresponds to the tissue repair phase. However, in the original study, the authors used the BBB score to assess the motor function of rats at four time points: the seventh, 14th, 21st, and 28th days after spinal cord injury. When using methods such as ELISA, Western blotting, immunofluorescence, transmission electron microscopy, HE staining and Evans blue staining to detect relevant indicators, the focus was only on the seventh day after injury. Therefore, we recommend including additional time points to better capture IRFV’s time-dependent efficacy and account for the dynamic pathophysiology.

### The optimal therapeutic concentration was determined through multi-dose IRFV studies

Additionally, we noted that the authors employed a single IRFV concentration (10 mg/kg) for *in vivo* administration without providing pharmacological justification for dose selection. Despite demonstrating efficacy in mitigating OGD/R injury to endothelial cells at 5 μg/mL *in vitro*, this concentration lacks correlation with the *in vivo* dosing regimen. Therefore, we recommend that the authors conduct *in vivo* multi-dose studies of IRFV to evaluate its therapeutic effects on SCI. In the study by Du Y et al., varying doses of berberine were administered to breast cancer model mice to determine its optimal anti-tumor dose based on changes in tumor growth and body weight (Du et al., 2022). We believe that this research can provide strong support for the suggestions we have put forward.

### The inadequacy of simplified *in vitro* models for recapitulating the BSCB microenvironment

This study employed an *in vitro* oxygen-glucose deprivation/reoxygenation (OGD/R) model with hCMEC/D3 endothelial cells to elucidate IRFV’s protection of the BSCB, demonstrating through Nrf2 inhibitor ML385 co-treatment that IRFV attenuates OGD/R-induced endothelial cell death and preserves barrier function; however, significant limitations undermine its physiological relevance. The *in vitro* model oversimplified the BSCB, which is a complex neurovascular unit (NVU). By using only hCMEC/D3 monocultures, it neglected critical components like astrocyte-derived induction, pericyte-mediated stabilization, and neuronal paracrine signaling (Leong et al., 2024). Furthermore, the OGD/R model does not model core SCI pathologies beyond ischemia, notably the inflammatory response, proteolytic disruption, and physical injury. Functionally, while Evans Blue extravasation and ZO-1/Occludin expression suggested permeability reduction, gold-standard quantifications like TEER (Transendothelial Electrical Eesistance) and SFP (Sodium Fluorescein Permeability Assay) were omitted (Jin et al., 2021). To enhance persuasiveness, we recommend:

1. Transwell co-culture of endothelial cells, astrocytes, pericytes and neurons was integrated to construct a more complete BSCB mode;
2. Some inflammatory factors were added to the model for pretreatment to simulate the inflammatory/proteolytic microenvironment (Santaguida et al., 2023; Nie et al., 2024);
3. Expanding functional assessment to TEER monitoring, SFP, and P-gp activity via Rho123 efflux—thereby establishing a clinically predictive BSCB platform.

### Defining IRFV’s specificity via Nrf2-KO: neuroinflammation and BSCB protection

As a master transcriptional regulator of cellular antioxidant defenses, Nrf2 mitigates inflammation and oxidative damage by transactivating cytoprotective genes (e.g., HO-1, NQO1). While this study demonstrated that (He et al., 2025): *in vitro* Nrf2 inhibition via ML385 abrogated IRFV’s therapeutic effects, and (Lee et al., 2019) *in vivo* IRFV significantly upregulated Nrf2/HO-1 expression in spinal cord injury models, definitive mechanistic validation through Nrf2-knockout (Nrf2^−/−^) systems remains lacking.

Furthermore, the author team might have overlooked some limitations of the Nrf2 inhibitor ML385. The study by Singh et al. (2016) pointed out that although ML385 is a commonly used Nrf2 inhibitor, it does not directly bind to the Nrf2 protein to inhibit its activity. In the complex cellular environment, it may bind to other unknown proteins, leading to unpredictable off-target risks. Therefore, it is even more necessary to construct an Nrf2 knockout model for further verification.

We believe that the research of Ni L et al. is worth learning (Ni et al., 2025). They identified the cardioprotective effect of exercise against TAC-induced injury to be mediated by Nrf2, given that Nrf2 knockout abolished the benefit. Therefore, we suggest that the author team subsequently detect inflammatory indicators such as IL-1b and Tnf-α and BSCB integrity indicators (such as TEER, Evans Blue extravasation) in the Nrf2 knockout model under IRFV intervention. A loss of IRFV efficacy in Nrf2-knockout models would confirm the pathway as essential for its protection, establishing causal inference and specificity.

### Advancing pharmacokinetic studies of IRFV is crucial for its development as a clinical therapy for SCI

The plant extract IRFV has advantages such as low toxicity and multi-target activity. The research of the author team provides a promising candidate drug for the development of new drugs for the treatment of spinal cord injury. The author clearly stated in the discussion section that the subsequent research work will further characterize the pharmacokinetic features of IRFV and determine the therapeutic effects of its key active ingredients. We affirm this idea and offer some specific methods that might be adopted:

1. Conducting systematic pharmacokinetic studies using LC-MS/MS (Liquid Chromatograph Mass Spectrometer) to quantify the time-dependent concentrations of IRFV and its major metabolites in plasma, cerebrospinal fluid, and spinal cord tissue.
2. Validating the contribution of key constituents through individual efficacy assessments. In addition, the dynamic change process of drug concentration in the injury area can be monitored in real time through spinal microdialysis technology.
3. Long-term toxicological experiments are conducted to assess whether IRFV has potential side effects on cells and tissues.

## Discussion

We commend the authors for their rigorous investigation, which innovatively elucidates how IRFV alleviates neuroinflammation and BSCB injury after SCI through Nrf2/HO-1 pathway activation. This work establishes a robust mechanistic foundation for combined antioxidant/anti-inflammatory therapies in SCI treatment. As a plant-derived compound, IRFV exhibits a favorable safety profile and cost-effectiveness, offering a viable alternative for developing novel SCI therapeutics. We anticipate future studies further delineating the pharmacodynamics of IRFV and the Nrf2/HO-1 signaling axis.

## References

[B1] Du Y. Khan M. Fang N. Ma F. Du H. Tan Z. (2022). Berberine attenuates cell motility *via* inhibiting inflammation-mediated lysyl Hydroxylase-2 and glycolysis. Front. Pharmacol. 13, 856777. 10.3389/fphar.2022.856777 35559258 PMC9086160

[B2] He Y. Lu J. Pang R. Ding L. Wang Y. Xiao H. (2025). Iridoids derived from Valeriana jatamansi jones alleviates neuroinflammation and blood spinal cord barrier permeability after spinal cord injury by activating the Nrf2/HO-1 signaling pathway. Front. Pharmacol. 16, 1597719. 10.3389/fphar.2025.1597719 40756981 PMC12313594

[B3] Jin L. Y. Li J. Wang K. F. Xia W. W. Zhu Z. Q. Wang C. R. (2021). Blood-Spinal cord barrier in spinal cord injury: a review. J. Neurotrauma 38 (9), 1203–1224. 10.1089/neu.2020.7413 33292072

[B4] Lee T. H. Hsieh S. T. Chiang H. Y. (2019). Fibronectin inhibitor pUR4 attenuates tumor necrosis factor α-induced endothelial hyperpermeability by modulating β1 integrin activation. J. Biomed. Sci. 26 (1), 37. 10.1186/s12929-019-0529-6 31096970 PMC6521375

[B5] Leong T. W. Gao Z. David E. T. Li X. Cai Q. Mwirigi J. M. (2024). Spatially precise and minimally invasive delivery of peptides to the spinal cord for behavior modulation. ACS Nano 18 (51), 34720–34729. 10.1021/acsnano.4c06030 39655357

[B6] Ni L. Lu B. Peng G. Ma W. Guo Z. Huang L. (2025). Exercise-preconditioning attenuates TAC-induced cardiac hypertrophy and myocardial injury through activating NRF2. Free Radic. Biol. and Med. 239, 80–90. 10.1016/j.freeradbiomed.2025.07.024 40681061

[B7] Nie X. Liu Y. Yuan T. Yu T. Yun Z. Xue W. (2024). Platelet-rich plasma-derived exosomes promote blood-spinal cord barrier repair and attenuate neuroinflammation after spinal cord injury. J. Nanobiotechnology 22 (1), 456. 10.1186/s12951-024-02737-5 39085856 PMC11290287

[B8] Santaguida S. Haghighat L. Stanimirovic D. B. (2023). Standardization of TEER measurement for blood-spinal cord barrier models. Fluids Barriers CNS 20 (1), 45. 10.1186/s12987-023-00455-0 37328833

[B9] Singh A. Venkannagari S. Oh K. H. Zhang Y. Q. Rohde J. M. Liu L. (2016). Small molecule inhibitor of NRF2 selectively intervenes therapeutic resistance in KEAP1-Deficient NSCLC tumors. ACS Chem. Biol. 11 (11), 3214–3225. 10.1021/acschembio.6b00651 27552339 PMC5367156

